# New 2D diffraction model and its applications to terahertz parallel-plate waveguide power splitters

**DOI:** 10.1038/srep41726

**Published:** 2017-02-09

**Authors:** Fan Zhang, Kaijun Song, Yong Fan

**Affiliations:** 1 EHF Key Laboratory of Science, School of Electronic Engineering, University of Electronic Science and Technology of China, Chengdu, Sichuan, 611731, P. R. China

## Abstract

A two-dimensional (2D) diffraction model for the calculation of the diffraction field in 2D space and its applications to terahertz parallel-plate waveguide power splitters are proposed in this paper. Compared with the Huygens-Fresnel principle in three-dimensional (3D) space, the proposed model provides an approximate analytical expression to calculate the diffraction field in 2D space. The diffraction filed is regarded as the superposition integral in 2D space. The calculated results obtained from the proposed diffraction model agree well with the ones by software HFSS based on the element method (FEM). Based on the proposed 2D diffraction model, two parallel-plate waveguide power splitters are presented. The splitters consist of a transmitting horn antenna, reflectors, and a receiving antenna array. The reflector is cylindrical parabolic with superimposed surface relief to efficiently couple the transmitted wave into the receiving antenna array. The reflector is applied as computer-generated holograms to match the transformed field to the receiving antenna aperture field. The power splitters were optimized by a modified real-coded genetic algorithm. The computed results of the splitters agreed well with the ones obtained by software HFSS verify the novel design method for power splitter, which shows good applied prospects of the proposed 2D diffraction model.

Geometrical optics (GO) and physical optics (PO), supplemented with geometrical theory of diffraction (GTD) and the uniform theory of diffraction (UTD) and the Huygens-Fresnel principle are usually applied in the analysis and synthesis of reflectors^1^,^2^. They are very powerful tools to solve the problems of reflector synthesis in 3D space. But they are difficult to be applied in 2D or quasi-2D condition due to the limitation of the boundary condition. Although both the conventional method-of-moment (MoM) and finite element method (FEM) provide a valid method to accurately calculate the field distribution in 2D, the electrically large size needs prohibitively large computer resources and leads to unacceptable time consumption of optimization. Thus, it is necessary to develop a 2D model that is simple but general and can be applied to design 2D and quasi-2D device.

In recent years, power splitter/combiner devices have been widely used in microwave and millimeter-wave system, such as the power combining amplifiers and the antenna arrays. Various power splitters/combiners, such as rectangular waveguide power splitters^3^,^4^,^5^, coaxial waveguide power splitters^6^,^7^, ring-cavity power splitters^8^,^9^, radial waveguide power splitters^10^,^11^, substrate integrated waveguide power splitters^12^,^13^, gap waveguide power splitter^14^ and 3D power splitters based on transformation optical theory^15^ have been studied. Especially, in order to apply the power combining technique to the high end of millimeter-wave band and THz frequency, several quasi-optical power combining techniques with the principle of holography performed in electromagnetic wavefront shaping attract more and more attention. Using the concept of hologram based on quasi-optical principle, multi-way holographic power splitters with high combining efficiency were presented^1^,^2^,^16^,^17^,^18^. However, either dielectric phase grating or 3D-shaped reflector are used in aforementioned holographic power splitters/combiners, resulting in huge size and difficulty in the assembly.

In this paper, a new 2D diffraction model is proposed and used to design terahertz parallel-plate waveguide power splitters in an easy and efficient way. The difficulty of designing these power splitters is how to achieve electromagnetic wavefront transformation with high efficiency. Here the shaped cylindrical reflectors are used that can be regarded as computer-generated holograms to transform one electromagnetic beam into multiple electromagnetic beams in a parallel-plate waveguide cavity. Moreover, we developed a modified real-coded genetic algorithm (MRGA) to solve the problem of shaping the reflector surface without gradient information. Compared with conventional design methods of power splitter, we believe that the method based on the 2D diffraction model in this work can be very useful for the shape optimization of power splitter.

## Methods

### Theoretical design

The Huygens-Fresnel principle^19^ can be used to describe the wave propagation from one surface to another in 3D free space. It can be expressed mathematically as follows:


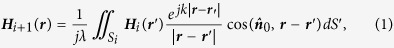


where ***H***_*i*+1_(**r**) is the magnetic field at the observation point at ***r*** · ***H***_*i*_(***r***’) and 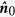 denote the magnetic field and the unit-normal vector at the integration point ***r***′ on surface *S*_*i*_, respectively. *k* = *ω/c* is wavenumber. 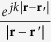 is the Green’s function of the Kirchhoff theory.

**Figure 1:**
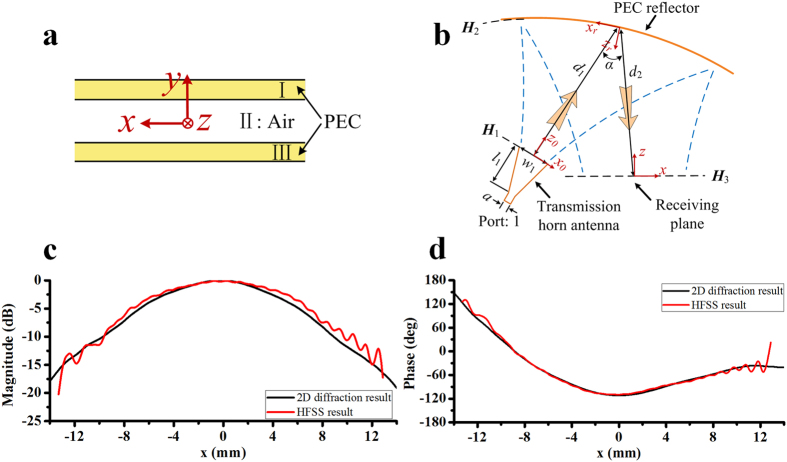
2D diffraction model. (**a**) Geometry of the 2D diffraction model. Section I and III are perfect electronic conductor (PEC) boundary. Electromagnetic beam exists in section II and it is in uniform distribution at *y* direction. (**b**) Schematics of a feed model with one reflector. The operating frequency is 300 GHz. *a* = 0.8636, *w*_1_ = 7, *l*_1_ = 7, *d*_1_ = 27, *d*_2_ = 25, (in mm) and *α* = 38°. The focal distance of the cylindrical paraboloid is 0.031 m. (**c**) Magnitude and (**d**) phase of normalized magnetic-field along the *x* axis at the receiving plane: 2D diffraction results (dark line), HFSS results (red line).

In order to state the Huygens-Fresnel principle, it is convenient to regard each point on the aperture as a new source of spherical waves. The result of our diffraction analysis can be regarded as the superposition integral in 3D free space. But in the 2D diffraction model shown in Fig. 1(a), the Huygens-Fresnel principle can be difficult to handle due to the boundary condition limits. And it is also not easy to strictly derive an analytical expression of the 2D diffraction model from Maxwell’s equations because of the complexity of the boundary conditions. In Fig. 1(a), section I and III are perfect electronic conductor (PEC) boundary. Section II is filled with air and its width is greater than its height. We consider that the electric field is y-polarized in section II and the field in the *y* direction to be uniform distribution, i.e., only relevant to the *x* and *z* directions. The field in the y direction is uniform distribution and only relevant to the x and z directions. So the diffraction in section II can be simplified into a 2D case. To rapidly and efficiently calculate the diffraction filed of section II, approximate analytical expression is offered by the analogy with the Huygens-Fresnel principle. The field in 2D diffraction model is calculated by equation (2).


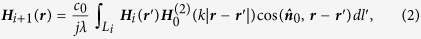


where 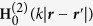 is the Bessel function of the third kind, and *k* = *ω/c* is wavenumber. 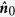 denotes the unit-normal vector on aperture surface (line) *L*_*i*_, *c*_0_ is complex constant. ***r**, **r***′ and 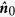 are only relevant to the *x* and *z* directions in the equation (2). Analogously, we regard the calculated filed as the superposition integral in 2D space. In order to verify the correctness of the proposed 2D diffraction model, an example of a 2D feed structure with one reflector is shown in Fig. 1(b). The H-plane sectoral horn antenna is as the transmission horn to radiate the y-polarized electromagnetic wave. Here we consider the y-polarized beam wave guidance and diffraction by a 2D reflector. The aperture field distribution of the H-plane sectoral horn antenna ***H***_1_ can be expressed in a local coordinate:


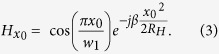


Here 
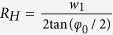
, 
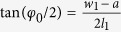
, 
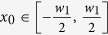
, *φ*_0_ is the flare angle of the H-plane sectoral horn antenna. In this paper, the time dependence of *e*^−*jωt*^ is assumed and suppressed.

In the 2D diffraction model there exists a cylindrical wave, the propagation and diffraction of cylindrical wave are calculated by the equation (2). ***H***_2_ is calculated by ***H***_1_ based on equation (2), and the diffraction field ***H***_3_ in the receiving plane can be obtained by ***H***_2_ from the equation (2) in Fig. 1(b). Figure 1(c) and (d) show the calculated magnitude and phase of normalized magnetic-field respectively, along with the values from an HFSS simulation for comparison. It can be see that the calculated results based on 2D diffraction model closely agree with the 3D full-wave electromagnetic simulation software HFSS ones based on the FEM.

**Figure 2:**
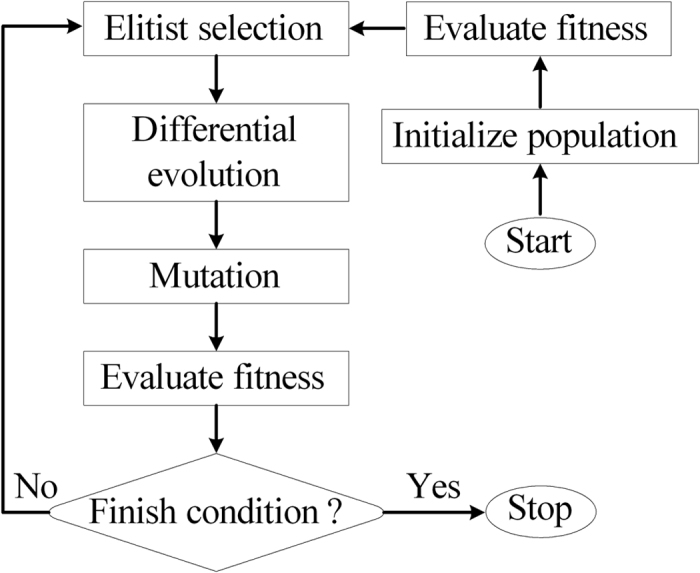
Flow chart of the MRGA.

In order to split a wide electromagnetic beam into many narrow beams, that is to say, serve as a power splitter, we have to shape the reflector. The optimization method is based on a modified real-coded genetic algorithm (MRGA). A differential evolution operator, which is borrowed from the differential evolution algorithm^20^ (DE), is introduced to, instead of the crossover operator^21^, obtain better global searching ability in the high density population environment. The process of MRGA can be represented by the flowchart form as shown in Fig. 2. The loop runs for a number for predefined generations and is composed mainly of selection, differential evolution, mutation, and evaluate fitness steps.

The first step is to realize the real coding of these problems. Here we give a detailed coding method for the algorithm. The initial shape of the reflector is cylindrical paraboloid and this surface can be expressed in the form:


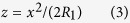


where *x* is along the local principal surface directions with *R*_1_ being its associated principal surface radii of curvatures.

We discretized the reflector surface into small strips with same period along the *x* coordinate. The *z* coordinate of all nodes of the surface can be expressed as a 1*N vector.


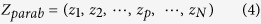


If each grid is variable, meaning, the surface is not smooth after the optimization is completed. Smooth procedures should be used to ensure the validity of the 2D diffraction and to ease the process of manufacture of the reflector with a milling machine. Here, an interpolation method is applied to smooth the reflector cylindrical surface in the real coding. A certain amount of nodes that are as the optimal variables is enough to interpolate all rest points. *Linear, spline* and *cubic* are several common interpolation methods in MATLAB. Interpolation method is a simple and efficient way to yield a smooth surface cylindrical reflector. The surface correction Δ*Z*_*interp*_ interpolates from all interpolating nodes Δ*Z*. The number of Δ*Z*_*interp*_ is about 3 times the number of Δ*Z* in this article. The actual cylindrical surface relief is


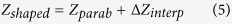


The Z_*shaped*_ will be used in the field calculation. Individuals Δ*Z* using real-coding can be expressed as below:


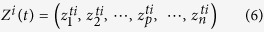


where 
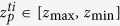
, *p* ∈ [1, *n*], *n* ≈ *N*/3, *i* ∈ [1, *NP*]. *NP* is the number of individuals in a population. *i* and *t* donate the *i*th individual and the *t*th generation, respectively. 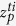 which is the interpolation node is as one of the variables *n* on the optimization and it is a real number. The variation range of the interpolation node 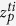 is from *z*_min_ to *z*_max_.

The next step is to define the loop of the optimization procedure. The individuals that take part in the reproduction step are determined using the roulette wheel method. It means that the individuals are assigned a probability of being selected based on their fitness. Especially, with the help of elitism, the best individual is stored into the next generation. In this way we cannot lose the best genetics.

Here we use differential evolution operator instead of crossover operator to obtain better global searching ability in the high density population environment. For each individual *Z*^*i*^(*t*), a perturbed individual *Z*^*i*^(*t* + 1) is generated according to scheme DE/best/1 20, the DE operator is expressed as below.


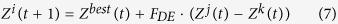


with *i, j, k* ∈ [1, *NP*], integer and *F*_*DE*_ is a real constant factor, 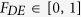. If 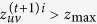, then let 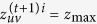, or if 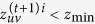, then let 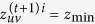, here u = 1.

Here, we used a random variation to exploit the solution space. For the *m*th mutation, 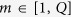, *Q* is the mutation number, we have


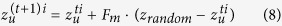


where 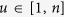 is the element index generated randomly and *z*_*random*_ ∈ [*z*_max_, *z*_min_].

If 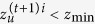 or 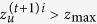, we have


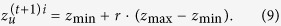


Where *r* is generated from the interval [0, 1]. The best performing individual in the (*t* + 1)th will not involve in the mutation and it will be preserved in the next generation in the selection operator.

Here we have defined a coupling coefficient *k* to characterize a potential solution as follows:


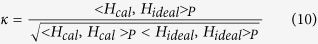


where *H*_*cal*_ and *H*_*ideal*_ are the calculated field and ideal field, respectively. They have the magnitude and phase information of the field. The scalar products for two complex-valued vector functions *f* and *g* are defined as


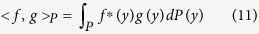


According to these definitions, it is easy to handle the complex-valued problem. The 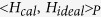 is normalized with 
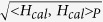
 and 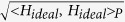 in order to guarantee the magnitude of the complex number |*κ*| in scale [−1,1]. If Re{*κ*} = 1, it means that the calculated field is same as the ideal one. So, the fitness function of the problem is defined as


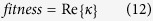


The objective is to maximize the fitness to get close to 1.

## Results

### THz 6-way power splitter with one shaped reflector

**Figure 3:**
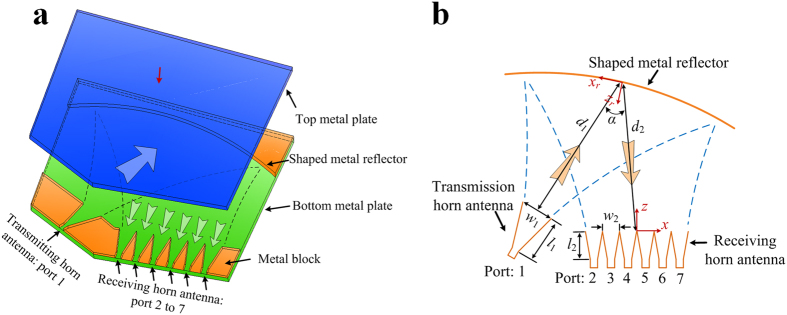
THz 6-way power splitter with one shaped reflector. (**a**) Schematics of a six-way power splitter with one shaped reflector. The power splitter is composed of three parts – the top metal plate, the cavity and the bottom metal plate. The distance between the two metal plates is 0.4318 mm, which is the narrow width of the standard rectangular waveguide WR3. (**b**) Simple top view of the power splitter. *w*_1_ = 7, *l*_1_ = 7, *d*_1_ = 27, *w*_2_ = 3.33, *l*_2_ = 6, *d*_2_ = 25, (in mm) and *α* = 38°. The focal distance of the original cylindrical paraboloid is 0.031 m.

**Figure 4:**
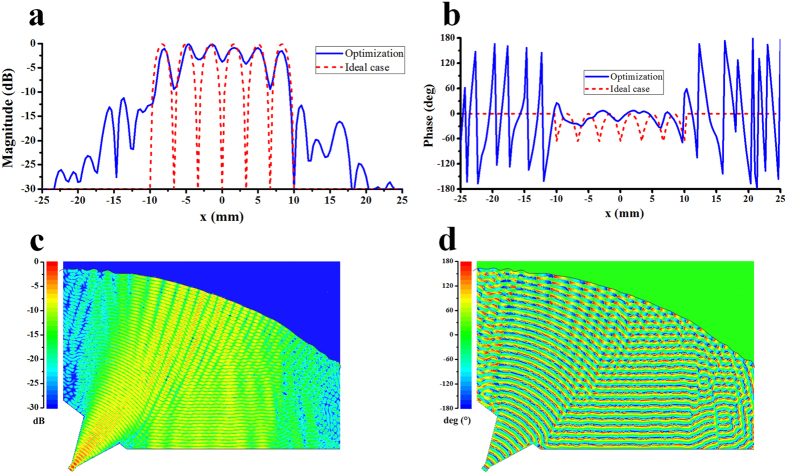
Optimized results of the 6-way power splitter without receiving horn antenna. (**a**) Magnitude and (**b**) phase of normalized magnetic-field at receiving plane along the *x* axis: ideal case (dashed line), optimized (solid line). (**c**) Magnitude and (**d**) phase of normalized electric-field at 300 GHz in the parallel-plate waveguide cavity by HFSS.

Figure 3(a) presents geometry of the proposed parallel-plate waveguide power splitter. Figure 3(b) is a top view of the power splitter. The power splitter takes electromagnetic beam from input port 1 and splits it into the six output ports 2–7 with the best possible performance of amplitude and phase balance. The beam splitting is achieved by the shaped metal reflector. The calculated results are obtained first at 300 GHz by Matlab programming based on equation (2) according to the above optimization procedure. To confirm the validity of this method, we import the optimized model into software HFSS based on FEM and create a 3D model to simulate. In the numerical calculation, the metal is regarded as PEC material. Figure 4(a) and (b) show the optimized magnitude and phase distribution of magnetic fields at 300 GHz on the receiving plane without receiving sectorial horn antenna by Matlab programming. The coupling coefficient *κ* = 0.89 + *j*0.01 is obtained through hundreds of generations of evolution by MRGA. Figure 4(c) and (d) show the distribution of the electric field and power flow of the power splitter with different transmission path in the cavity by software HFSS. It can be seen that the beam has been split into six narrow beams at receiving plane.

**Figure 5:**
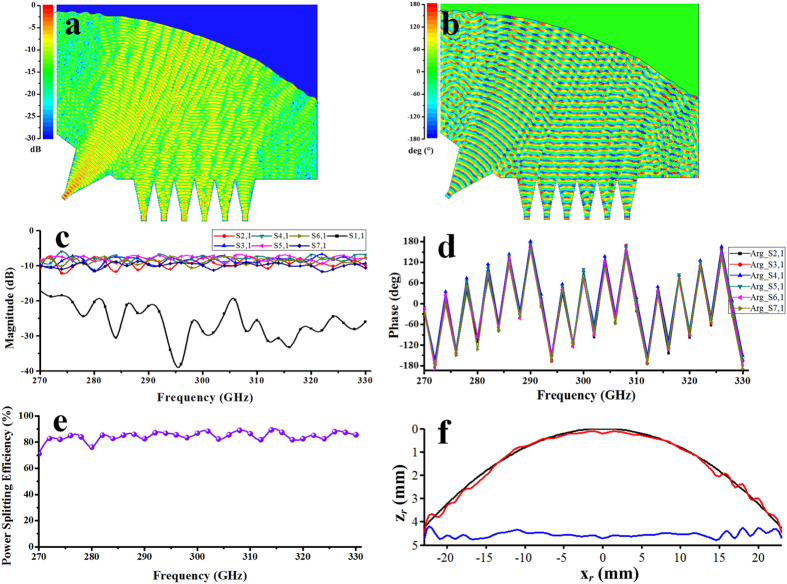
Performance of the 6-way power splitter with receiving horn antenna. (**a**) Magnitude and (**b**) phase of normalized electric-field in the parallel-plate waveguide cavity by software HFSS. (**c**) *S*-parameters of six-way power splitter with receiving horn antenna. (**d**) Phase characteristic. (**e**) Power splitting efficiency of the power splitter. (**f**) Shaped surface relief: initial surface relief (dark line), superimposed surface (blue line) and actual surface (red line).

Figure 5(a) and (b) show the magnitude and phase distribution of electric fields at 300 GHz with receiving sectorial horn antenna array in the cavity. The ideal cases of magnitude and phase are calculated by equation (3). Figure 5(c) shows the reflection coefficient and transmission coefficient obtained by the discrete sweep type of software HFSS with 2 GHz interval. The reflection coefficient is related to S11. A low magnitude of S11 is desirable and results in a lower reflection. In Fig. 5(c), the magnitude of S11 is less than −17 dB over the whole bandwidth, which means that almost all energy is fed into the parallel-plate waveguide cavity from port 1 and very few energy comes back to port 1. Transmission coefficient is related to the magnitude of Sn1 (in this case, n = 2,…, 7) denoting the amount of energy transmits from port 1 to port n. The magnitude of the Sn1 is around −8.6 dB (the −7.8 dB power division loss for a 6-way splitter is include). The ripples of the magnitude of Sn1 are caused by the field mismatch and multiple reflections between the transformed field and the ideal receiving antenna aperture field. One possible solution to reduce the ripples is to design a less sensitive field matching structure such as gap waveguide technology compared with conventional H-plane receiving horn antenna. Good phase characteristic can be obtained over the whole bandwidth in Fig. 5(d). The phase imbalance is ±13.1 degrees at 300 GHz. Figure 5(e) shows the power splitting efficiency over the whole bandwidth with 20% relative bandwidth. It is noted that the theoretic average power splitting efficiency excited by the dominant mode TE_10_ from input port 1 reaches to 83.5% in the case of leakage of beam wave from both sides of the power splitter. There are reasons to believe that the higher-order modes are weak and do not affect the performance of the power splitter greatly. Considering the metallic losses of ordinary brass using a value of conductivity of 3 × 10^7^ S/m and 5 μm surface roughness, the average power splitting efficiency degrade from 83.5% (PEC materials) to 57.6%. We should also notice that the ohmic loss can be greatly reduced by the selection of high conductivity materials and the decrease of the model size due to the shorter transmission path. The shaped reflector, i.e., the computer generated hologram, can be seen in Fig. 5(f). In this case, the surface is composed of 139 nodes. The number of the optimized nodes is 47 of 139 nodes. Using the 47 optimized nodes to interpolate all 139 nodes to get a smooth superimposed surface profile. The superimposed surface Δ*z*_*r*_ is obtained using the interpolation function *spline (x*_*r*_, Δ*z*_*r*_) of MATLAB. It provides the piecewise polynomial form of the cubic spline interpolant to the data values Δ*z*_*r*_ at the data sites *x*_*r*_. The actual surface expressed by *z*_*shaped*_ = *z*_*parab*_ + Δ*z*_*r*_, i.e., the shaped diffracting surface profile, is the surface relief superimpose Δ*z*_*r*_ upon the initial surface *z*_*parab*_.

### THz 10-way power splitter with two shaped reflectors

**Figure 6:**
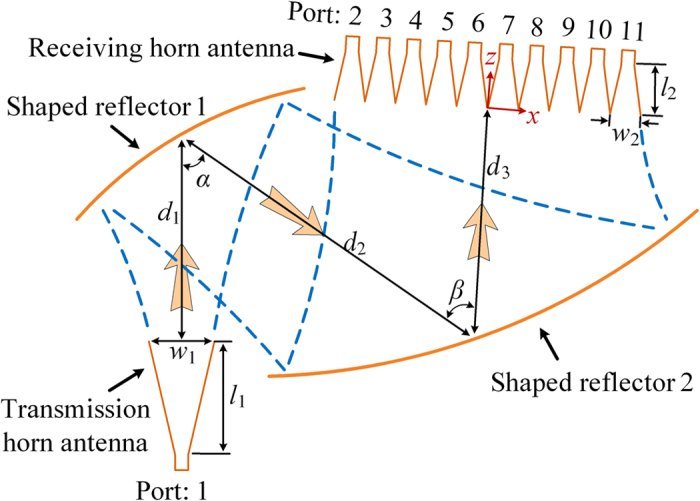
THz 10-way power splitter with two shaped reflectors. Simple top view of the power splitter with two shaped reflectors. The power splitter is also composed of three parts – the top metal plate, the cavity and the bottom metal plate. The distance between the two metal plates is 0.4318 mm, which is the narrow width of the standard rectangular waveguide WR3. *w*_1_ = 6.66, *l*_1_ = 12.3, *d*_1_ = 25, *w*_2_ = 3.33, *l*_2_ = 6, *d*_2_ = 39.6, *d*_2_ = 26.6 (in mm) and *α* = 54°, β = 58°. The focal distance of the first original cylindrical paraboloid is 0.0467 m, and the second is 0.075 m.

**Figure 7:**
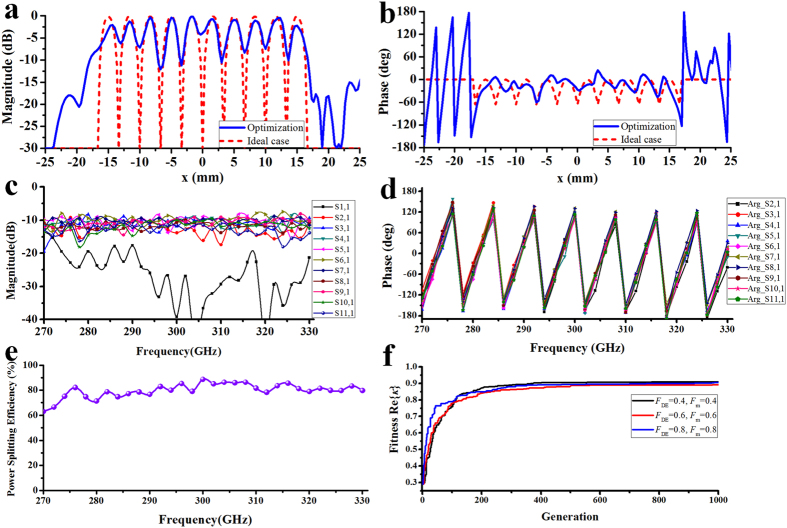
Results of the THz 10-way power splitter with two shaped reflectors. (**a**) Magnitude and (**b**) phase of normalized magnetic-field without receiving horn antenna along the *x* axis: ideal case (dashed line), optimized (solid line). (**c**) *S*-parameters of 10-way power splitter with receiving horn antenna. (**d**) Phase characteristic. (**e**) Power splitting efficiency of the power splitter and (**f**) convergence characteristics of the MRGA.

The top view of the 10-way parallel-plate waveguide power splitter is shown in Fig. 6. It is clear that two reflectors add degrees of freedom to optimization and more ways beam splitting can be realized. Figure 7(a) and (b) show the optimized 10-way magnitude and phase distribution of magnetic fields at 300 GHz on the receiving plane without receiving sectorial horn antenna by Matlab programming. The coupling coefficient *κ* = 0.91 + *j*0.00 is obtained through one thousand generations of evolution by MRGA. Figure 7(c) shows the transmission coefficient obtained by the discrete sweep type of software HFSS with 2 GHz interval. Good phase characteristic can be obtained over the whole bandwidth in Fig. 7(d). The phase imbalance is ±15.5 degrees at 300 GHz. Figure 7(e) shows the power splitting efficiency. It is noted that the theoretic average power splitting efficiency reaches to 79.8% (PEC materials). Figure 7(f) shows the progress of the MRGA optimization as the function of the number of generations with different *F*_*DE*_ and *F*_*m*_. It takes about 21 minutes to complete a single trail. In all trials, the number of individuals *NP* = 4 and the mutation number of each reflector surface is fixed *Q* = 2. The numerical results demonstrate that the MRGA runs well with high efficiency and stability. It shows that design method based on MRGA reached the same goal through different routes.

## Discussion

We have developed a 2D diffraction model that is economic and efficient numerical method able to compute electrically large multireflector power splitter. This method, 2D diffraction model, works with a modified real-coded genetic algorithm, however, unlike conventional MoM and FEM, has good performance in electrically large size splitter. Two real-world design tasks appearing in the development of power combining technique have been solved successfully by applying the 2D diffraction model. They are good candidates for exciting an array of slot antennas or the design of planar grid amplifiers. Two reflectors add degrees of freedom to optimization and more ways of power splitter can be realized with good results. It should be noted that the proposed terahertz parallel-plate waveguide power splitter offers the following advantages that include: 1) quasi-planar and compact, 2) operating at millimeter to THz wave, 3) multiway, 4) simple design and assembly.

## Additional Information

**How to cite this article**: Zhang, F. *et al*. New 2D diffraction model and its applications to terahertz parallel-plate waveguide power splitters. *Sci. Rep.* **7**, 41726; doi: 10.1038/srep41726 (2017).

**Publisher's note:** Springer Nature remains neutral with regard to jurisdictional claims in published maps and institutional affiliations.
